# Mannose: A Sweet Option in the Treatment of Cancer and Inflammation

**DOI:** 10.3389/fphar.2022.877543

**Published:** 2022-05-13

**Authors:** Fang Nan, Yutong Sun, Hantian Liang, Jingyang Zhou, Xiao Ma, Dunfang Zhang

**Affiliations:** Department of Biotherapy, State Key Laboratory of Biotherapy and Cancer Center, West China Hospital, Sichuan University, Chengdu, China

**Keywords:** mannose, treg cells, autoimmune disorders, tumor therapy, mannose phosphate isomerase, glycolysis

## Abstract

As a natural sugar, mannose is a type of hexose that is abundant in many different types of fruits. Since mannose is rarely used for glycolysis in mammals, studies on the role of mannose have not attracted much attention. Glycosylation of specific proteins was thought to be the major function of mannose. Surprisingly, during the past few years, mannose was found to be effective in promoting immune tolerance and suppressing inflammatory diseases related to autoimmunity and allergy. Moreover importantly, mannose was also found to be efficient in suppressing tumors by suppressing glycolysis and enhancing chemotherapeutic agents. In this review, we summarize the recent studies of mannose on antitumor properties and anti-inflammatory characteristics. We emphasize that mannose could play a beneficial role in the treatment of a variety of diseases, including cancers and inflammatory diseases, and could be a novel therapeutic strategy that deserves continued evaluation.

## Introduction

In recent years, the general public has paid more and more attention to the relationship between diet and disease. Adjusting diet has also been shown to enhance cancer treatment (Kanarek et al., 2020; Tajan and Vousden, 2020). Among the hexoses people consume on a daily basis, mannose appears to be the most attractive in many ways. Mannose is a hexose, which is a component of many polysaccharides. Mannose is poorly metabolized in the human body, so it is not obvious that mannose enters carbohydrate metabolism after oral administration. Mannose plays an important role in human metabolism, especially in glycosylation of certain proteins (Alton et al., 1998) involved in immune regulation. Congenital glycosylation disorder (CDG) is a kind of disorder that is difficult to diagnose clinically due to its various manifestations and abnormal functions of multiple organ systems (Jaeken and Péanne, 2017; Péanne et al., 2018). CDG-Ib (MPI-CDG) is caused by mutations in mannose phosphate isomerase (MPI) (Freeze and Sharma, 2010). Based on the fact that MPI deletion can lead to liver fibrosis (Janssen et al., 2014), DeRossi et al. conducted experiments with zebrafish as a model, and observed that MPI deletion in zebrafish larvae can lead to liver development defects (DeRossi et al., 2019). They demonstrated that acute and persistent MPI deletions are sufficient to activate hematopoietic stem cells and induce fibrosis *in vitro* and in whole vertebrate models, suggesting that MPI deletions promote stellate cell activation. Direct production of mannose-6-phosphate based on mannose phosphorylation circumvented the MPI defect (Niehues et al., 1998; Lonlay and Seta, 2009), and together with experimental results, showed that mannose reduced stellate cell activation and fibrosis.

During the last few years, mannose has been shown to have many physiological benefits and applications, such as suppressing inflammation (Zhang et al., 2021), and suppressing tumor growth and metastasis (Gonzalez et al., 2018). Besides suppressing Type 1 diabetes, asthma and macrophage-mediated inflammation (Zhang et al., 2017; Torretta et al., 2020), a most recent study has shown that mannose can suppress experimental autoimmune encephalomyelitis (EAE) by inducing treg cells, suggesting that Mannose may be a potential adjuvant therapy for multiple sclerosis (MS) (Hwang et al., 2021). More importantly, mannose has been determined to be effective in suppressing different kinds of cancers (Gonzalez et al., 2018). This review focuses on the research progress of mannose in treating tumors and inflammation. We emphasize the potential of mannose as a therapeutic agent for the above-mentioned diseases and raise questions to be addressed.

## The Anticancer Effects of Mannose

### Mannose Interferes With Glucose Metabolism of Tumor Cells

**FIGURE 1:**
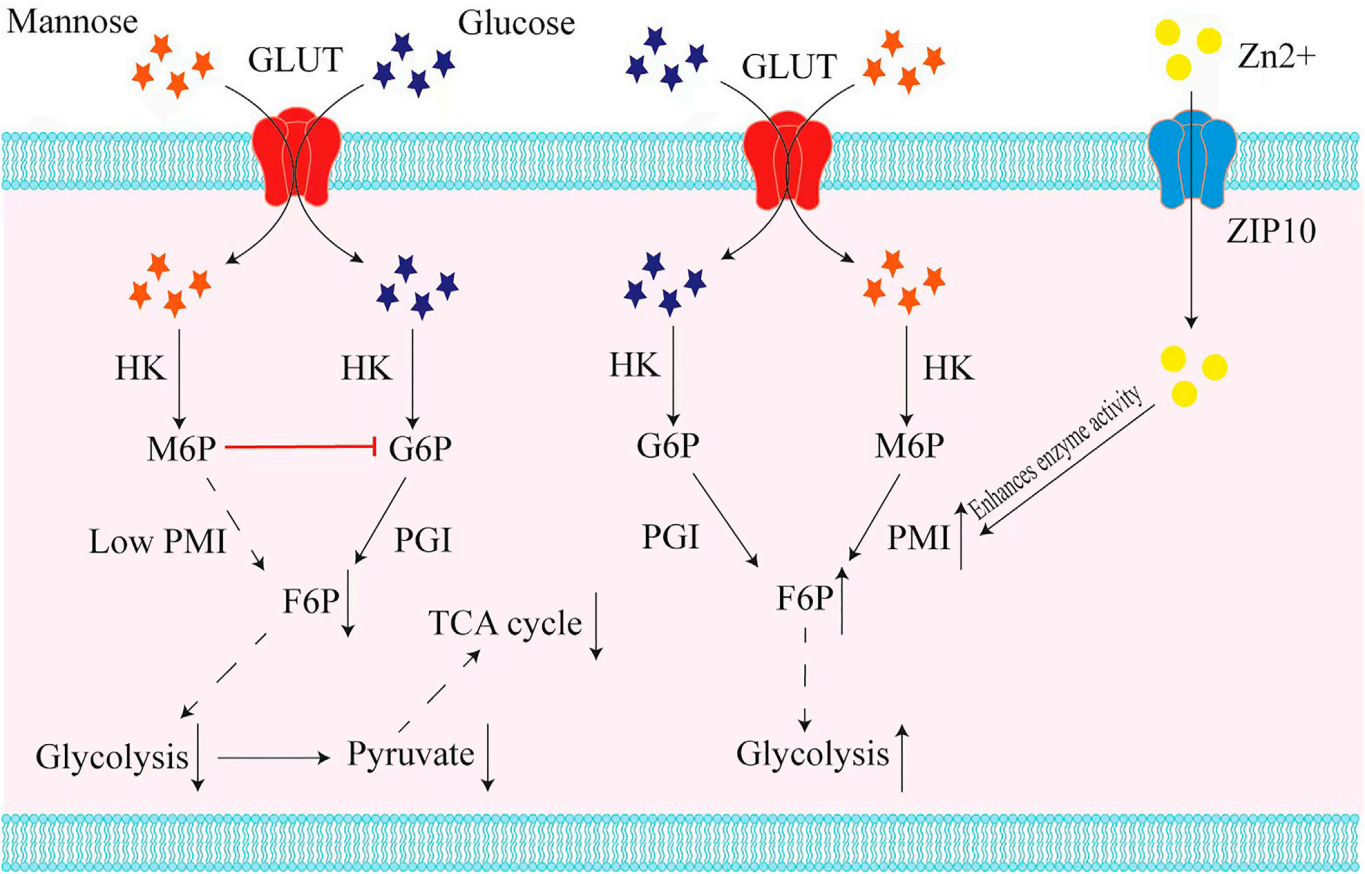
Mannose interferes with glucose metabolism in tumor cells with low expression of the MPI gene. Mannose and glucose are transported into cells by the same transporter (GLUT), and transformed by hexokinase (HK) into mannose-6-phosphate (M6P) and glucose-6-phosphate (G6P). In those tumor cells with low mannose phosphate isomerase (MPI) expression, the accumulation of M6P suppresses glucose phosphate isomerase (GPI) and other enzymes related to glucose metabolism, resulting in inhibition of glycolysis and in turn inhibition of the tricarboxylic acid cycle (TCA). In contrast, in tumor cells that express a high level of MPI, mannose could be used for glycolysis efficiently. Recent studies have found that Zn^2+^ is crucial for mannose phosphate isomerase (PMI) to exert enzymatic activity, and when ZIP10, a transporter of Zn^2+^, is knocked down, it causes the inactivation of MPI and makes tumor cells become mannose sensitive.

Tumor cells usually have a high demand and affinity for glucose (Bhattacharya et al., 2016). Based on the fact that mannose and glucose are introduced into cells by the same transporters (Thorens and Mueckler, 2010), Gonzalez et al. have proposed that mannose may interfere with glucose uptake by tumor cells, and made relevant experimental verification (Gonzalez et al., 2018). Interestingly, the results showed that mannose treatment did not reduce or even increase the intracellular glucose level, but affected cell growth by interfering with glucose metabolism (Gonzalez et al., 2018). The mechanism at hand is that mannose accumulates in the form of mannose-6-phosphate and the accumulation of M6P suppresses glucose phosphate isomerase (GPI) and other enzymes related to glucose metabolism, which impairs the further metabolism of glucose in glycolysis, tricarboxylic acid cycle, pentose phosphate pathway, and glycan synthesis (Gonzalez et al., 2018). In follow-up experiments, they came up with two important results: First, among various hexoses, only mannose-combined with conventional chemotherapy such as cisplatin and adriamycin can down-regulate Mcl-1 and Bcl-XL protein levels and enhance apoptosis; Second, the inhibitory effect of mannose was negatively correlated with the expression level of mannose phosphate isomerase (PMI) encoded by MPI gene (Figure 1). Therefore, cells with low PMI levels are sensitive to mannose, while cells with high levels are resistant. These results could open up a novel way to fight cancer and provide a safe, simple, potential treatment strategy effectively against many types of cancer by enhancing chemotherapy. However, in terms of the effective dose of mannose, we expect scientists to calculate an appropriate dosage that has maximum effectiveness without any significant side effect. Based on the above mechanisms, a recent study has shown that mannose inhibits cell proliferation and enhances radiation-induced apoptosis in human esophageal squamous carcinoma cells with low MPI expression in a dose-dependent manner (Luo et al., 2022). This suggests that mannose can act as a radiation sensitizer in patients with esophageal squamous cell carcinoma with low MPI expression. There is also a study showed that in the early and middle stages of the development of glioblastoma multiforme (GBM), mannose can be combined with temozolomide concurrent radiotherapy (RT/TMZ) to achieve cure (Liu et al., 2020).

### Inhibitory Effect of Mannose on Various Tumors

For thyroid cancer, one recent study has found that whether the cell line is sensitive to mannose depends mainly on the enzyme activity of PMI (Ma et al., 2021). Structurally, zinc ions (Zn^2+^) are critical for the binding of the PMI and substrate, so the enzymatic function of PMI depends on Zn^2+^ concentration (Bangera et al., 2019). ZIP10 is a Zn^2+^ transporter expressed during early B cell development that transports Zn^2+^ from outside the cell to the cytoplasm, thereby altering the concentration of Zn^2+^ within the cell (Miyai et al., 2014). The latest study found that in thyroid cancer cells that are not sensitive to mannose, knocking down ZIP10 can significantly improve their sensitivity to mannose, and M6P accumulation in turn enhances the inhibitory effect of mannose on cellular glycolysis (Ma et al., 2021) (Figure 1). These findings suggest that mannose has potential application value in the treatment of thyroid cancer.

**FIGURE 2:**
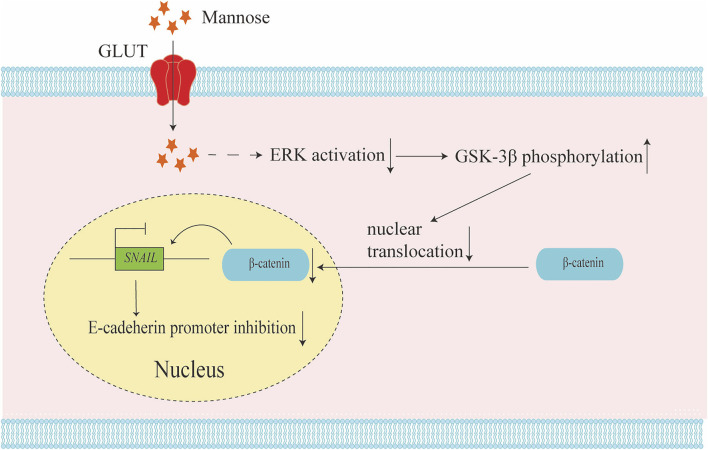
Mannose inhibits tumor metastasis by relieving epithelial-mesenchymal transition (EMT). Mannose could reduce the activation of the ERK pathway and increase the phosphorylation of GSK-3β. The phosphorylation of GSK-3β will reduce the phosphorylation level of tyrosine 654 at β-catenin and inhibit the nuclear translocation of β-catenin. The reduction of β-catenin in the nuclear down-regulates SNAIL expression, and the down regulation of SNAIL could promote E-cadherin expression. This regulatory mechanism results in the inhibition of tumor EMT.

In non-small cell carcinoma (NSCLC), it has been proved that mannose has a significant inhibitory effect on the proliferation, invasion, and metastasis of A549 cells. Mannose was shown to be able to induce G0/G1 arrest by down-regulating the expression of PCNA and up-regulating the expression of p21, both of which are related to the concentration of mannose (Wang et al., 2020). Moreover, mannose can promote the expression of phosphorylated GSK-3β, suppress the activation of ERK, and thereby reduce the expression of β-catenin and SNAIL, which in turn reduce N-cadherin and increases E-cadherin, thereby suppressing tumor metastasis (Wang et al., 2020; Luo et al., 2020) (Figure 2). In terms of mechanism, epithelial-mesenchymal transition (EMT) plays an indispensable role in cancer metastasis (Heerboth et al., 2015). Several transcription factors are upregulated in EMT metastatic cells, represented by SNAIL. SNAIL can be combined with the E-cadherin promoter to suppress the expression of E-cadherin and promote EMT (Al Khatib et al., 2019). Phosphorylation of GSK-3β and phosphorylation and destabilization of β-catenin have significant effects on EMT (Geng et al., 2017; Tian et al., 2020). The activation of ERK promotes the phosphorylation level of β-catenin Tyr654, and the phosphorylation of tyrosine 654 at β-catenin promotes nuclear translocation, which in turn promotes the expression of β-catenin in the nucleus. The phosphorylation of GSK-3β can suppress the function of β-catenin and promote the degradation of β-catenin. Mannose significantly reduces the phosphorylation level of tyrosine 654 at β-catenin in cells *in vitro* by reducing the phosphorylation level of ERK, and promotes the expression of phosphorylated-GSK-3β (Luo et al., 2020). Wang et al. also found that mannose could exert anticancer effects on A549 and H1299 cells by suppressing PI3K/AKT and ERK signaling pathways (Wang et al., 2020). This implies that mannose can be combined with AKT or ERK targeted therapy to treat lung cancer, but further experiments are needed to explore the molecular mechanism of mannose’s anti-cancer effect. It is worth noting that the suppressed migration induced by mannose is more effective on the migration of carboplatin-treated A549 cells. The last point is that mannose significantly enhances the effect of apoptosis after carboplatin treatment (Sha et al., 2020; Wang et al., 2020). All these findings suggest that mannose may be a selective cancer treatment. However, some problems remain, such as in chronic myeloid leukemia (CML), although D-mannose treatment can inhibit K562 cell growth *in vitro*, it does not reduce the tumor volume *in vivo*, and ponatinib and D-mannose treatment show severe drug toxicity in mice (Okabe et al., 2021). This reminds us that some approaches require more evaluation when it comes to targeted therapy for glucose metabolism.

### Targeting Mannose Receptors to Construct a Liposome System

\Some peptides act as regulators of innate immunity, activating macrophages and monocytes, thereby releasing chemicals with potential tumor-killing effects (Kager et al., 2010). As an excellent carrier of peptide drugs, liposomes can protect the structure and biological activity of drugs (Kumar Giri et al., 2016). Tumor epitope peptides can be recognized by dendritic cells (DCs) and processed into MHC-I complexes to specifically activate CD8^+^ T cells into cytotoxic T cells (CTLs) to achieve targeted killing effects (Wculek et al., 2020). The mannose receptor (MR) is a member of the C-type lectin superfamily, which is mainly present on the membrane surface of macrophages and DC cells, and plays an important role in antigen presentation (Martinez-Pomares, 2012). Therefore, recently, a relevant research team has developed a mannose modified liposome-epitope peptide drug delivery system, in which mannose improves the bonding rate of liposomes and DC cells, improves the targeting efficiency of epitope peptide liposomes to DC cells, and significantly inhibits the hematogenic spread of lung metastasis of triple negative breast cancer (TNBC) in animal experiments, and has good biosecurity (Yu et al., 2021). In addition, a research team has developed a doxorubicin (DOX)-based mannose nanogel (DM NG), which induces immunogenic cell death (ICD), stimulates the phagocytosis of DC cells, and promotes specific T cell anti-tumor immunity (Ma et al., 2022). The study also showed that the mannose released by DM NG interferes with glucose metabolism and thus regulates tumor metabolism, ultimately leading to apoptosis of cancer cells. It is also pointed out that this strategy can systematically modulate the tumor microenvironment (TME) and improve the immunosuppressive tumor microenvironment (ITM).

## The Anti-inflammatory Effects of Mannose

Besides the anticancer effects, mannose has been proven to suppress inflammation by suppressing glycolysis, promoting TGF-β activation and inducing Treg cells (Zhang et al., 2017; Torretta et al., 2020). Since it has been well summarized in the recent past that mannose is a novel suppressor of inflammation (Zhang et al., 2021), we only emphasize the point that mannose treatment could be a safe and promising strategy to suppress and to treat autoimmunity and asthma. More than that, mannose could also suppress wounding-induced inflammation in skin (Jokela et al., 2013). Hyaluronic acid (HA) is the only non-sulfated glycosaminoglycan in the extracellular matrix (Genasetti et al., 2008). It is involved in endothelial cell proliferation, migration, neovascularization, and leukocyte recruitment, and its metabolism is significantly altered in inflammation (Tammi et al., 2005). During inflammatory stimulation, the HA receptor,CD44, on the surface of leukocytes binds to the HA cable structure (La Motte et al., 1999; La Motte et al., 2003). *In vitro*, it has been reported that mannose consumes UDP GlcNAc to reduce the synthesis of HA (Jokela et al., 2008). Tiina et al. found that mannose also reduced the content of HA *in vivo* and prevented the binding of leukocytes to HA on the cell surface (Jokela et al., 2013). Not only that, they also found IL-1β induces the HA shell in keratinocytes to reconstitute the cable structure, and mannose can inhibit the binding of HA dependent monocytes to keratinocytes. They also found that mannose could suppress the function of dermal fibroblasts, and this potential anti fibrosis effect needs to be verified by further *in vivo* experiments.

## Conclusion and Future Perspective

Mannose is used in a variety of daily processes mainly because of its low heat and non-toxic characteristics (Wu et al., 2019). It also has the potential for clinical application in drug therapy (Wei et al., 2020). Mannose has shown anti-tumor effects against a variety of tumors, and it is safe in humans at certain concentrations (Gonzalez et al., 2018). In terms of dosage, a person would need to ingest very large amounts of mannose to match the amounts that were given to the mice in the experiments. Therefore, it is critical to find a way to effectively use mannose for human disease treatment.

In addition, related studies have shown that mannose is a biomarker of insulin resistance, which may help in the early identification of patients with insulin resistance and who are more prone to diabetic complications (Ferrannini et al., 2020). It was also found that plasma mannose levels increased in patients with insulin resistance independently of obesity (Mardinoglu et al., 2017). They confirmed that elevated plasma mannose levels were a strong marker for the risk of chronic diseases such as cardiovascular disease and type 2 diabetes and may contribute to their development, although the exact mechanism remains unclear (Mardinoglu et al., 2017). These evidence shows that mannose might have important applications in disease screening and detection.

In conclusion, mannose therapy is expected to be a promising strategy for the treatment of cancer and inflammatory diseases, although it is still needed to fully elucidate the mechanism and accurately evaluate and determine its effective dose in humans.
